# Bactericidal Silver Nanoparticles by Atmospheric Pressure Solution Plasma Processing

**DOI:** 10.3390/nano10050874

**Published:** 2020-05-01

**Authors:** Janith Weerasinghe, Wenshao Li, Rusen Zhou, Renwu Zhou, Alexander Gissibl, Prashant Sonar, Robert Speight, Krasimir Vasilev, Kostya (Ken) Ostrikov

**Affiliations:** 1 School of Chemistry and Physics, Queensland University of Technology, Brisbane 4000, Queensland, Australia; sonar.prashant@qut.edu.au (P.S.); kostya.ostrikov@qut.edu.au (K.O.); 2 Centre for Materials Science, Queensland University of Technology, Brisbane 4000, Queensland, Australia; 3 School of Biology and Environmental Science, Queensland University of Technology, Brisbane 4000, Queensland, Australia; wenshao.li@hdr.qut.edu.au (W.L.); alexander.gissibl@qut.edu.au (A.G.); robert.speight@qut.edu.au (R.S.); 4 School of Mechanical, Medical and Process Engineering, Queensland University of Technology, Brisbane 4000, Queensland, Australia; rusen.zhou@hdr.qut.edu.au; 5 School of Chemical and Biomolecular Engineering, The University of Sydney, Sydney 2006, New South Wales, Australia; renwu.zhou@sydney.edu.au; 6 School of Engineering, University of South Australia, Adelaide 5001, South Australia, Australia; krasimir.vasilev@unisa.edu.au

**Keywords:** silver nanoparticles, AC – DBD plasma, plasma production of nanoparticles

## Abstract

Silver nanoparticles have applications in plasmonics, medicine, catalysis and electronics. We report a simple, cost-effective, facile and reproducible technique to synthesise silver nanoparticles via plasma-induced non-equilibrium liquid chemistry with the absence of a chemical reducing agent. Silver nanoparticles with tuneable sizes from 5.4 to 17.8 nm are synthesised and characterised using Transmission Electron Microscopy (TEM) and other analytic techniques. A mechanism for silver nanoparticle formation is also proposed. The antibacterial activity of the silver nanoparticles was investigated with gram-positive and gram-negative bacteria. The inhibition of both bacteria types was observed. This is a promising alternative method for the instant synthesis of silver nanoparticles, instead of the conventional chemical reduction route, for numerous applications.

## 1. Introduction

Silver (Ag) nanoparticles have been widely studied due to their applications in a broad area of industries, such as biomedical engineering, textiles, healthcare, electronics, optics, and chemical engineering [1,2,3,4]. A number of synthesis techniques have been developed for the generation of silver nanoparticles [5]. Chemical reduction is one of the most popular techniques that employs reducing agents, such as ascorbic acid, to reduce the silver from its precursor solution [6]. However, thorough water treatment processes are required to remove the unused reducing agents from the final product since some reducing agents, such as hydrazine and sodium borohydride, are toxic [2]. Nanoparticle agglomeration occurs due to the high active surface area and capping agents, such as Poly (Viny Pyrrolidone) (PVP) or Poly (Vinyl Alcohol) (PVA), which are used to prevent the agglomeration of the formed nanoparticles [4]. A number of other methods are also used for the silver nanoparticle synthesis such as biosynthesis, photochemical and sonochemical pathways [3,4]. These techniques still require either several processing steps or time to obtain pure yield of the synthesised product. Here, we address these issues and demonstrate a versatile synthesis of functional silver nanoparticles by atmospheric pressure solution plasma processing.

Atmospheric pressure plasmas, which can be generated in low temperature and non-equilibrium conditions, are commonly referred to as cold plasmas [7,8,9,10]. Unlike low-pressure plasmas, expensive vacuum systems and pumps are not required for the cold atmospheric plasma generation. Several cold atmospheric plasma devices are available, such as Dielectric Barrier Discharge (DBD) plasma sources, microwave plasma systems, plasma jets, plasma arrays, etc. Of these systems, plasma sources based on DBD are versatile and are commonly used [8]. These are generally powered with AC power supplies within the range of 50 Hz to a few hundred kHz. A wide range of gases can be employed in these systems, such as Helium (He), Argon (Ar), Nitrogen (N_2_), and air. The highest plasma density has been reported in microwave plasmas and the electron temperature of the plasmas can reach several eV [8]. A typical plasma discharge contains charged species, energetic photons and radical species [10]. Interactions between the cold plasma and different materials lead to diverse reactions. The main classes of these reactions are plasma etching, plasma-assisted deposition, and plasma treatment processes (functionalisation) [7,11]. The depth of the plasma surface functionalisation is only about a few nanometers, while the depth of etching could be up to hundreds of nanometres and the films of micrometre and larger thickness can be deposited [10].

Among the many reactive species generated by plasma–liquid interactions, solvated electrons are of particular interest because they are highly reactive and can reduce a number of cations, anions and neutral species [12]. The nanoparticles of noble metals (Au, Ag, Pd, Pt), other metals (Ni, Cu, Zn, Sn), metal alloys and metallic compounds (MoS_2_, ZnMgS), metallic oxides (CuO, ZnO, TiO_2_), and nano composites have been produced by solution plasma processing [13].

Several experimental setups have been reported for the plasma–liquid synthesis of Ag nanoparticles [14]. Parameters such as precursor medium, electrode configuration, and electric power sources were varied in these studies. The production of silver nanoparticles, using an aqueous electrochemical cell with a micro plasma cathode and a solid metal anode was demonstrated by Richmonds et al. [11]. Kondeti et al. have performed Ag nanoparticle synthesis using Radio Frequency (RF) plasma jet by using an Ar and H_2_ gas mixture [1]. Thai et al. used Alternative Current (AC) glow discharge plasma to produce spherical nanoparticles. They were able to synthesise nanoparticle sizes varying from three to a few hundred nanometres in a spherical shape [3].

In the current study, a simple-to-use, single-step approach based on atmospheric pressure DBD plasma is used to synthesise stabilised Ag nanoparticles within a few minutes of time. This synthesis method does not require any added reducing agents and only stabiliser agents were used with the silver precursor. The effects of the plasma exposure time and the chemical changes occurring during the treatment were studied. Ultraviolet-visible (UV-vis) spectroscopy, transmission electron microscopy (TEM), and zeta sizer instruments were used to characterise the synthesised Ag nanoparticles. We also characterised the DBD plasma using optical emission spectroscopy (OES) studies, and the antibacterial effect of the synthesised Ag nanoparticles was also investigated. This method is scalable, environmentally friendly and versatile.

## 2. Experimental Methods

**Figure 1:**
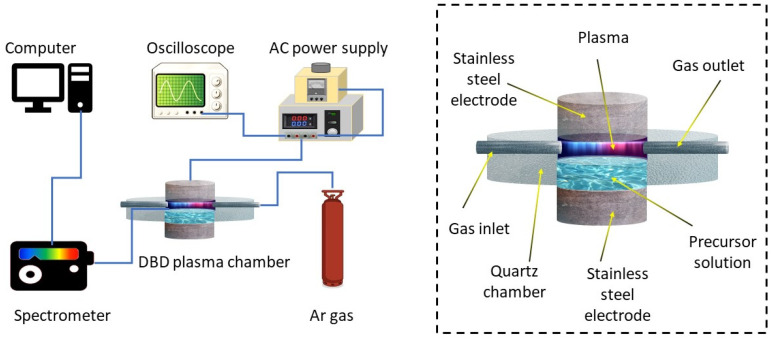
Schematic diagram of the DBD plasma setup used for the Ag nanoparticles synthesis. Inset: detailed diagram of the DBD plasma system.

Figure 1 shows a schematic diagram of the DBD plasma reactor used for this work. The reactor is a cylindrical structure made of quartz. The silver precursor solution was prepared by mixing 1.2 mM of aqueous Silver Nitrate (AgNO_3_) solution with 34 mM of Sodium Citrate solution in the ratio of 1:3 *v*/*v*. The hollow centre part of the plasma chamber could be filled with the precursor solution and could be covered with a quartz lid. The centre part was connected to the gas feeding line and the outlet. The total volume of the chamber is 25 mL and during the experiment 4 mL of the precursor solution was used. The lid and the bottom of the quartz chamber act as the dielectric barriers, with their thickness being 2 mm and the distance between the dielectric barriers being 8 mm. A high voltage was applied to the stainless-steel electrodes by CTP 2000K power supply and the amplitude and frequency were 30 kV and 9.1 kHz, respectively. Voltage and current measurements were made using Rigol DS6104 oscilloscope.

Optical emission spectroscopy (OES) were conducted using an Andor SR-500 spectrometer, Newton CCD camera and fibre optic cable (Oxford Instruments plc, Oxon, UK). The absorption spectra of the resulting solutions were recorded on a UV-Vis spectrometer (Cary 60 UV-Vis Spectrophotometer, Agilent Technologies Inc., Santa Clara, CA, USA). Transmission electron microscopy (TEM, JEOL 2100, JEOL Ltd., Tokyo, Japan) was used to analyse the geometry and size distribution of the nanoparticles. During the sample preparation, an aqueous solution of suspended nanoparticles was added dropwise on carbon-coated copper TEM grids and dried under ambient conditions overnight. A selected area electron diffraction (SAED) ring pattern was used to calculate the crystal spacings using the reported procedure [15]. Lattice fringe spacing was determined by the profile plot of Image J software. Malvern Zetasizer Nano ZS (Malvern Panalytical Ltd., Malvern, UK) used for the Dynamic Light Scattering (DLS) measurements to determine the size distribution. Glass cuvettes were used as sample holders and the samples were filtered through “Hydraflon” hydrophilised PTFE filters with pore size of 0.22 µm before the data acquisition. The measurement parameters are: material refractive index 1.40, Dispersant Refractive index 1.330, and measurement temperature 25 °C.

For the antibacterial studies, a modified version of the Mueller-Hinton method was used [16,17]. 6-mm Whatman^®^ filter papers and Luria-Bertani (LB) broth and agar were supplied by Sigma-Aldrich Pty Ltd., Castle Hill, NSW, Australia. The *E. coli* DH5α strain was purchased from New England Biolabs Ltd., Ipswich, MA, USA. *Staphylococcus aureus* ATCC^®^ 25923™ was purchased from Thermo Fisher Scientific Pty Ltd., Waltham, MA, USA. *E. coli* and *S. aureus* were cultured on LB agar plates and single colonies were used to inoculate 10 mL of LB broth and grown at 37 °C until the optical density (OD) at 600 nm reached 0.6. A total of 200 μL of the liquid culture was evenly spread on nutrient agar plates. A total of 10 μL of each silver nano particle sample exposed to different plasma exposure times was added to the centre of a 6-mm filter paper disk prior to placement on the inoculated LB agar plates and incubated overnight at 37 °C to observe zones of bacterial growth inhibition.

## 3. Results and Discussion

### 3.1. Particle Formation and Characterisation

**Figure 2:**
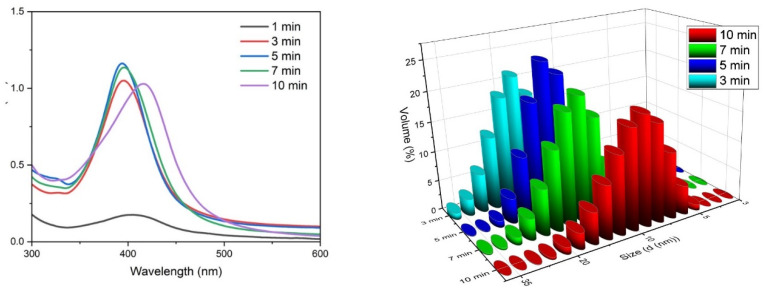
Ultraviolet-visible (UV-Vis) spectra of the silver nanoparticles synthesised on different plasma exposure times (**left**); size distribution of Ag nanoparticles measured by the Dynamic Light Scattering (DLS) method technique for different plasma exposure times (**right**).

The colour of the precursor solution changed to yellow after a few minutes of the plasma treatment, indicating the formation of the nanoparticles. All the samples present the characteristic surface plasmon resonance peak of silver nanoparticles (Figure 2). A slight but significant peak was observable for 1 min of plasma exposure, but this later became more prominent. The width of the each plasmon is related to the size distribution of the nanoparticles. The 3-min, 5-min and 7-min samples produced peaks at 369 nm, 394 nm and 396 nm, respectively. The 1-min and 10-min samples have their surface plasmon peaks at 404 nm and 416 nm, respectively. Since equal volumes of samples reacted using the same concentration of precursor, the peak height of the absorbance can be directly proportional to the concentration of the synthesised nanoparticles. The nanoparticle concentration increases until it reaches 5 min and then there is a slight decrease at 7 min. At 10-min plasma exposure, the decrease is more prominent and the plasmon resonance peak is red shifted, which can be attributed to polydisperse distribution of silver nanoparticles [18].

Unlike microscopic techniques, DLS measures the hydrodynamic diameter of the theoretical sphere (rather than the physical size) that diffuses with the same speed as the measured nanoparticle. This is determined by the stabilisers adsorbed on to the nanoparticle, and the solvation shell also moves along with the particle [19]. Therefore, the size measured using the DLS technique is slightly larger than the results obtained by the microscopic techniques. Moreover, DLS measurements are hardly possible on smaller particles in a polydisperse solution [20]. According to the DLS data obtained from the samples (Table 1 and Figure 2 right), there is a decrease in nanoparticle size with an increase in plasma exposure time. The largest average diameter of 20.06 nm is observed at 3 min of plasma treatment and it was then observed that the diameter decreases gradually with an increase in treatment time. The lowest average particle diameter of 9.99 nm was observed at 10 min of plasma exposure. This is an important finding since sub 10-nm silver nanoparticles has been proven to be extremely effective on antibacterial activity. Since DLS measures the hydrodynamic diameter of the nanoparticles, the actual size of the resulting particles can be much smaller than 9.99 nm, which will be far more effective on disinfection processes. Additionally, more than 90% of the volume percentage can be found within the peak area, indicating a narrow distribution of nanoparticles formed during the experiment.

**Figure 3:**
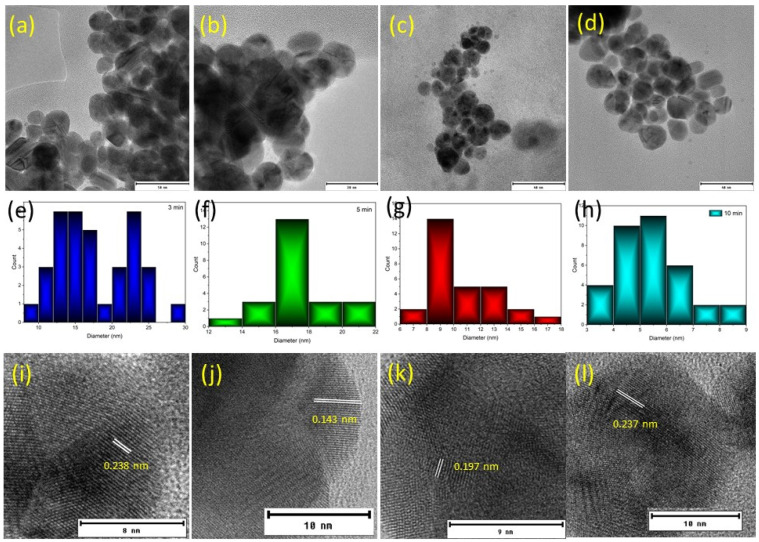
Transmission Electron Microscopy (TEM) images of the nanoparticles synthesised under different plasma exposure times: (**a**) 3 min, (**b**) 5 min, (**c**) 7 min, (**d**) 10 min; size distribution of the nanoparticles determined from TEM: (**e**) 3 min, (**f**) 5 min, (**g**) 7 min, (**h**) 10 min; HRTEM of nanoparticles (**i**) 3 min, (**j**) 5 min, (**k**) 7 min, (**l**) 10 min.

The morphology of the silver nanoparticles has been analysed using TEM. Figure 3a–d show the images of the particles synthesised under 3, 5, 7 and 10 min of cold plasma exposure. The shape is near spherical and the average diameters of the nanoparticles were 17.9 ± 8.9, 14.3 ± 1.6, 10.6 ± 2.3, and 5.4 ± 1.4 nm, respectively. The High-resolution transmission electron microscopy (HRTEM) images of the silver nanoparticles synthesised under different plasma exposure times are shown in the Figure 3e–g.

The fringe spacing is measured to be 0.235, 0.14 and 0.202 nm, which corresponds to the (111), (220) and (200) planes of silver. The d spacings are 0.236, 0.220, 0.141 and 0.121, indicating the face-centred cubic (fcc) crystal structure. The results are consistent with the previous reports in literature [5,21,22]. The sizes obtained on these samples match with the data obtained in the above DLS measurements.

### 3.2. Antibacterial Properties

**Figure 4:**
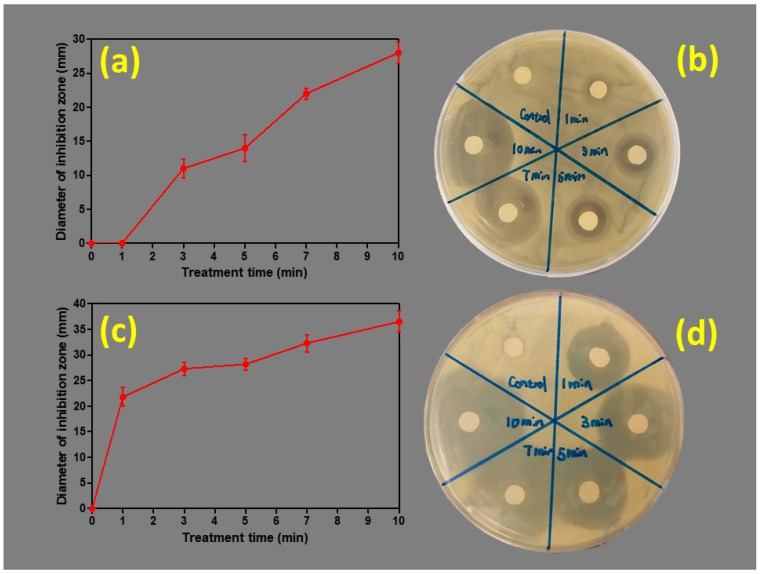
Treatment (plasma exposure time) time curves for (**a**) *E. coli* and (**c**) *S. aureus*. Example optical image of *E. coli* clearance zones (**b**) and *S. aureus* (**d**) on agar plates with different silver nanoparticle samples under visible light.

Equal volumes of nanoparticle suspensions were infused onto the filter paper disks that were added to bacterial plates and the growth inhibition zones were observed after incubation for 24 h. The variation in the size of the inhibition zones was shown to depend on the plasma exposure (treatment) time, as shown in Figure 4. The diameter of the growth inhibition zone was observed to increase when the plasma exposure (treatment) time was increased. *S. aureus* was seen to be more sensitive to the silver nano particles compared to *E. coli*. Based on the UV-Vis data, there was a slight difference in the silver nanoparticle concentrations in the 5-min, 7-min and 10-min plasma-treated samples. However, this antibacterial study showed an inverse relationship between the silver nanoparticle size and the antibacterial activity (Figure 4). These observations align with previous studies where decreased particle size increased the antibacterial effect of silver nanoparticles [23,24,25,26].

Silver nanoparticles are capable of increasing concentrations of reactive oxygen species and decreasing reactive nitrogen species, which cause oxidative stress in bacteria [27,28]. Anti-fungal and anti-viral activities are also reported for silver nanoparticles [29]. The attachment of nanoparticles to the cell wall and cell membranes damages the cell wall and internal structures and causes changes to biochemical pathways. Properties of the nanoparticles, such as size, shape, surface coating and surface charge, also affect the antibacterial activity [30]. *S. aureus* inhibition by silver nanoparticles has been reported to occur through cell wall damage [31]. For *E. coli*, a variety of toxic effects have been reported upon exposure to silver nanoparticles, including respiratory inhibition, plasma membrane depolarisation, the leakage of intercellular potassium ions, and metabolic activity inhibition [32].

### 3.3. Mechanism of Nanoparticle Formation

**Figure 5:**
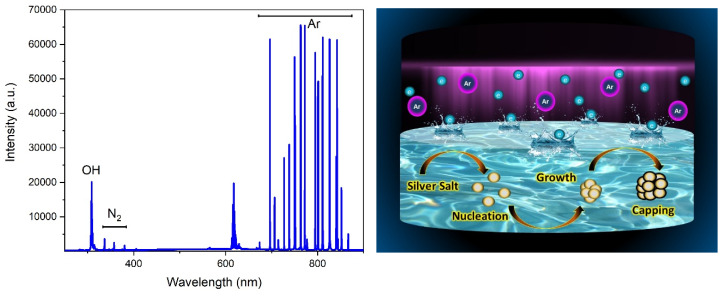
OES spectrum of the Argon DBD plasma used to treat the precursor solution (**left**); schematic representation of the DBD plasma assisted silver nanoparticle formation (**right**).

The mechanism of nanoparticle formation is related to the species generated by the plasma and is illustrated in Figure 5 (right). The chemical reactions taking place during the experiment occur in three different regions. These are the plasma region, plasma–liquid interface and liquid region. Species present in the plasma are excited atoms, ions, electrons and UV photons. In this experiment, Ar DBD has been used and, hence, the main energy carriers for the generation of reactive species are excited Ar atoms and few nitrogen species. The origin of nitrogen species may be due to the dissolved nitrogen in the solution or impurities of the gas. Species such as OH, O_3_, and H_2_O_2_ can also be generated due to the presence of water vapour from the aqueous precursor solution. Optical emission spectroscopy (OES) measurements were performed to identify the species in the plasma discharge. This analyses the light emissions from the constituents of the plasma, such as neutral or ionised gas atoms, free radicals and other molecules. Figure 5 (left) shows the emission spectra of the plasma, which is dominated by argon bands. The hydroxyl band (A^2^Σ to X^2^ Π, 0 - 0, 1 - 1, 2 - 2), at 309 nm [33], can be identified and would originate due to the dissociation of water. The second positive system emission of N_2_ (C^3^Π_µ_ to B^3^Π_g_) can be seen in low intensity at 337, 357 and 380 nm. The nitrogen lines may occur due to the dissolved nitrogen in the aqueous precursor solution. Moreover, oxygen emission lines can be found in 777 nm and 844 nm. Identified spectral lines for argon are included in Table 2 including transitions, relative intensities, and energy levels. 

The transfer of these species occurs in the plasma–liquid interface and a fraction of these species is transported into the liquid interface. Within the gas–liquid interface, the possible reactions to take part are the recombination, absorption or desorption of reactive species and the solvation of the electrons [34]. Due to the complex mechanisms of these chemical reactions, a number of unexplained questions remain regarding the plasma-induced liquid chemistry.

The solvated electrons absorbed into the liquid penetrate a few nanometers and react with solvent molecules to induce a number of chemical reactions [12] inclusive of the reduction of silver ions Equation (1). The redox potential of Ag^+^/Ag is (+0.799 V), which makes the instant capture of the solvated electrons and synthesis of the reduced silver atoms. The atmospheric pressure plasma may have electrons with an energy level up to 20.2 eV and it is possible for the positive ions to also have energy levels above 10 eV [3]. The following reaction is possible within the few nanometres of the liquid surface.

e_aq_ + Ag^+^ → Ag (1)

Furthermore, solvated electrons react with water and H^+^ to form more H, which reacts with silver ions to reduce them to Ag Equations (2) and (3). These reactions form H, which also contributes to the further reduction of silver ions.

e_aq_ + H^+^ → H (2)

e_aq_ + H_2_O → H + OH (3)

The synthesis of silver nanoparticles in the liquid occurs by the nucleation of the reduced metal atoms due to the liquid chemistry initiated by the DBD plasma. According to the OES spectra, excited Ar, OH and N_2_ species were identified. The reduction could occur due to a number of species, such as plasma electrons, hydrogen radicals, solvated electrons and hydrogen peroxide [14]. Thai et al. reported that fragments of water continuously ionised to produce OH^+^, O^+^, and H^+^ because the ionisation energies are located below the energy threshold. Additionally, the reaction between H and silver cations also forms silver metal atoms by the following reaction in Equations (2) and (3), which also facilitates the formation of Ag seeds.

H + Ag^+^ →Ag + H^+^ (4)

Nanoparticle growth occurs eventually by the combination of metallic atom clusters which are surrounded by the citrate capping agents. According to Equations (1)–(4), the amount of reducing agents required to form silver ions depends on the exposure time, and the reaction kinetics can be controlled by varying it. According to the TEM images, the size and the size distribution of the synthesised nanoparticles depend on the plasma exposure. This phenomenon can be explained using the La Mer’s model [2,35]. On low plasma exposure duration, the reducing species in the solution are scarce and few nuclei are formed for the nucleation step. As such, only particle growth can be observed with a broader size range. When the exposure time increases, the enhanced reduction rate generates more nuclei, which leads to the formation of silver nanoparticles of a smaller size. The amount of the reactants available for the particle growth step decreases due to the large number of nuclei, and a relatively smaller number of nanoparticles can be observed in a narrow size distribution.

## 4. Conclusions

Silver nanoparticles were successfully synthesised by DBD solution plasma processing. Our results suggest that the plasma exposure time is an important factor for the resulting silver nanoparticle concentration and morphology. The morphology was characterised by Transmission Electron Microscopy (TEM) and the optical properties were determined by ultraviolet and visible spectroscopy. The DLS technique was used to determine the hydrodynamic diameter of the particles. OES measurements were made to identify the species present in the cold plasma. The antibacterial properties of the synthesised silver nanoparticles were studied using *S. aureus* as a model of gram-positive bacteria and *E. coli* as a gram-negative bacterium. The results indicate that the synthesised nanoparticles demonstrated increased anti-microbial activity with decreasing nanoparticle size. This work demonstrates the possibility of producing silver nanoparticles without the need for toxic reducing agents. The resulting nanomaterials can be successfully employed in electronics, plasmonic and biological applications. The same technique can be used for the synthesis of other various metal nanoparticles, removing the need for chemical reducing agents, leading to much more economically and environmentally favozurable processes.

## Figures and Tables

**Table 1:** Variation of DLS size values on different exposure times of cold plasma.

Plasma Exposure (min)	DLS (nm)	% Volume	Standard Deviation
3	20.06	90.9	5.543
5	16.16	95.9	3.746
7	15.27	92.2	4.322
10	9.99	98.4	3.405

**Table 2:** Observed optical emission spectroscopy (OES) data of the argon emission spectral lines. Transition, initial energy levels (Ei) and final energy levels (Ef) taken from Ref [10].

Wavelength (nm)	Transition	Relative Intensity (a.u)	E_i_ (eV)	E_f_ (eV)
696	2p_2_ → 1s_5_	61,371	13.33	11.55
707	2p_3_ → 1s_5_	15,587	13.30	11.55
715	2p_5_ → 1s_5_	3284	13.28	11.55
727	2p_2_ → 1s_4_	26,821	13.33	11.62
738	2p_3_ → 1s_4_	30,896	13.30	11.62
750	2p_1_ → 1s_2_	55,719	13.48	11.83
751	2p_5_ → 1s_4_	36,434	13.27	11.62
763	2p_6_ → 1s_5_	65,549	13.17	11.55
772	2p_2_ → 1s_3_	65,450	13.15	11.55
795	2p_4_ → 1s_3_	57,756	13.28	11.72
801	2p_6_ → 1s_4_	48,875	13.09	11.55
810	2p_7_ → 1s_4_	50,477	13.15	11.62
811	2p_9_ → 1s_5_	62,028	13.08	11.55
826	2p_2_ → 1s_2_	61,613	13.33	11.83
841	2p_3_ → 1s_2_	27,474	13.30	11.83
842	2p_8_ → 1s_4_	61,277	13.09	11.62
852	2p_4_ → 1s_2_	18,474	13.28	11.83
